# Dosimetric absorption of intensity-modulated radiotherapy compared with conventional radiotherapy in breast-conserving surgery

**DOI:** 10.3892/ol.2014.2704

**Published:** 2014-11-12

**Authors:** YANG LIN, BENZHONG WANG

**Affiliations:** 1 Department of Radiation Oncology, The First Affiliated Hospital of Anhui Medical University, Hefei, Anhui 230022, P.R. China; 2 Department of Breast Surgery, The First Affiliated Hospital of Anhui Medical University, Hefei, Anhui 230022, P.R. China

**Keywords:** breast cancer, intensity-modulated radiotherapy, breast-conserving surgery, radiotherapy

## Abstract

The aim of this study was to investigate the dosimetric benefits between intensity-modulated radiotherapy (IMRT) and conventional radiotherapy (CR) among patients receiving breast-conserving surgery. A dosimetric comparison of IMRT and CR was evaluated in 20 patients with early-stage breast cancer using a three-dimensional treatment planning system. The prescribed mammary gland dose was completed in 25 fractions with a total dose of 5,000 cGy. Homogeneity of the planning target volume (PTV), irradiation dose and volume of organs at risk (OARs) were evaluated through a dose-volume histogram. For the homogeneity of PTV, the average volume receiving 95% of the prescribed dose in the IMRT plan was similar to that in the CR plan (97 vs. 96%, respectively). With regard to normal tissue sparing in OARs, the ipsilateral lung V_20_ in the IMRT and CR plans was 27.8 and 20.8%, respectively. The mean dose and V_30_ of the heart for five patients were 598.4 versus 348.3 cGy and 10.06 versus 5.3%, respectively. The mean dose sparing the heart or lung was markedly reduced in the IMRT plan compared with the CR plan. The results of the current study demonstrated that whole breast IMRT improves PTV dose distribution and improves normal tissue sparing in OARs.

## Introduction

Breast-conserving surgery may eradicate macroscopic diseases that have been detected and palpated in females with early-stage breast cancer (1,2). However, certain microscopic tumor foci may remain in the conserved breast, leading to local recurrence and/or life-threatening distant metastases. The administration of adjuvant radiotherapy following breast-conserving surgery is effective in reducing the risk of locoregional recurrence and distant metastases in patients with early-stage breast cancer (1,2). Postoperative radiation treatment in patients with breast cancer is conventionally delivered using external beam radiation therapy, which is determined by rectangular tangential fields. With this radiotherapy technique, an appreciable dose within the irradiated volume may be administered, and the dose delivered to the lung and heart may be higher than predicted (3).

Over the past decade, there has been a rapid increase in the utilization of advanced radiation delivery technologies for the curative management of numerous types of solid cancer. Radiation patterns have shifted from conventional two-dimensional (2D) radiotherapy to a more developed three-dimensional (3D) approach in treating breast cancer (4,5). However, whether intensity-modulated radiotherapy (IMRT) is superior to traditional 3D radiation delivery remains unknown.

In recent years, the use of IMRT has been greatly improved, as the beam intensity profile has been conformed to the chest wall or delineated target volume, resulting in reduced radiation dose variations and sparing of organs at risk (OAR)(6–9). The shape of the IMRT plan can be optimized on the basis of geometrical parameters, including the shape of the breast and thoracic wall, or dosimetric parameters using inverse planning. Consequently, it has not always been possible to establish a satisfactory compromise between the dose delivered to the target volume or the clinical tumor volume (CTV) and the dose delivered to the OARs (10,11). Compared with conventional rectangular tangential fields, the dose distribution conforms more to the target volume when 3D data are available and conformal treatment fields are used. This approach reduces the dose to OARs. Intensity modulation may be considered as an additional step and allows for greater freedom in improving the dose distribution compared with the combination of open and wedged beams. This may result in a further improvement of the dose distribution in the target volume and the OARs. Inverse planning provides a method for minimizing the dose to OARs, whilst maintaining adequate target coverage.

The majority of IMRT studies have shown a potential clinical benefit in sparing OARs and improving dose homogeneity over the target volume, as compared with rectangular tangential fields without conformal blocks (12–14). However, the implementation of IMRT in clinical practice requires additional resources for patients with breast cancer in the adjuvant setting, as the IMRT plan is more time-consuming and complex. Therefore, it is useful to identify patients with a medical necessity for the IMRT plan. Several studies have compared a number of forms of IMRT, including forward and inverse methods, with conventional radiotherapy (CR) (15–17). However, the relative improvement attributable to intensity modulated irradiated fields compared with conformed fields is not yet known for the irradiation of patients with early-stage breast cancer. Therefore, a clinical trial was initiated to investigate radiation dosimetry of IMRT, assessed by changes in breast appearance and discomfort in patients with early stage breast cancer.

## Patients and methods

### Eligibility

A total of 20 patients under the care of the Department of Radiation Oncology, the First Affiliated Hospital of Anhui Medical University (Hefei, China), with early-stage breast cancer (T_1–2_N_0_M_0_; stage I or II_A_), according to 7th edition of American Joint Committee on Cancer (18), between July 2008 and October 2009 were included in this study. Patients with no previous malignancies, complete microscopic excision of tumors and histological confirmation of breast cancer underwent breast-conserving surgery (16 patients with invasive ductal carcinoma, two with invasive lobular carcinoma, one with intraductal carcinoma, and one with medulla carcinoma). Radiotherapy was prescribed for the whole breast, and written informed consent was obtained prior to the trial. The study was approved by the ethics committee of The First Affiliated Hospital of Anhui Medical University, and was conducted in accordance with the declaration of Helsinki.

### Patient positioning and localization

Localization of the treatment volume and the field geometry was conducted by an Acuity™ simulator (Varian Medical Systems, Inc., Palo Alto, CA, USA) according to laser mark points on the bodies of the patients. The computed tomography (CT) results were applied to this retrospective treatment planning study. CT slices were acquired every 5 mm, with the patient lying in a supine position. Patients were positioned in the supine position on an angled board with the arms abducted to 90°, such that the sternum was horizontal. All patients were positioned with the arms resting on an armrest placed above the head equivalent to the treatment position. The patients were immobilized about the shoulders and upper arms, by applying a vacuum air cushion across the shoulders. This position has been demonstrated to facilitate treatment planning of tangential fields without the arms extending into the treatment fields and changing the shape of the breast significantly (19). The CT scan included the complete left and right lung, breast, heart and liver. The median separation distance between the most medial and lateral aspects of the breast was 21.1 cm (range, 18.0–26.5 cm), which was confirmed in the current study. All graphic files of the CT scans were transmitted to the Topslane treatment planning system (Xops 2.0; Shanghai Topslane Medical Technology Co., Ltd., Shanghai, China) for further analysis.

### Target volume outline

The CTV was delineated by a radiation oncologist according to surface mark points and CT scan files, which were optimized to visualize the glandular tissue of patients that had received breast-conserving surgery. The radiation oncologist contoured the CTV based on the CT scan. The CTV was assumed to start 3–5 mm below the skin and delineated smoothly with an anterior margin of 0.5 cm beneath the skin, a posterior margin of the chest wall surface, a medial margin of the body midline and a lateral margin of midaxillary line. A planning target volume (PTV) was generated by expanding the CTV by 7 mm isotropically, with the exception of the direction towards the skin surface, where expansion stopped at 5 mm below the skin, to account for the uncertainty in the patient set-up and CTV delineation. The cranial extent of the heart included the infundibulum of the right ventricle, right atrium and right atrium auricle, but excluded the pulmonary trunk, ascending aorta and superior vena cava. The lowest external contour of the heart was the caudal border of the myocardium, with the pericardium excluded. The contours of the lungs and skin were automatically outlined.

### CR plan

The gantry and collimator angles of the tangential fields were selected using the beam’s eye view option on the 3D treatment planning system (3DTPS; Xops 2.0). The edges of the tangential fields were non-divergent, to minimize the irradiated lung volume to a margin of 1.5–2.0 cm. In the conformal plans, an automatically generated conformal wedge block around the PTV, with a margin of 1.5–2.0 cm, was added to these fields. The treatment planning was performed using the 3DTPS (Xops 2.0). The optimum wedge angle and beam weights were calculated using the optimization module. In the context of an equivalent beam weight of the tangential fields, the goal of the optimization module was to obtain a homogeneous dose, while maintaining a low dose in the lungs and heart.

### IMRT plan

Using the same gantry angles as applied in the CR plans, tangential 6-MV photon beam intensity profiles were calculated using the inverse planning program (radio-SOFT 1.0; Apache Technologies Inc., Dayton, OH, USA). Following sequencing, the segment weights were refined with the optimization module using the same objective score function as the CR plan. From the PTV to normal tissues, the final dose distribution was calculated to inversely optimize the beam intensity profiles, which in turn was converted to a segmental sequence. Thus, the same dose calculation algorithm was used for all rectangular, conformal and step-and-shoot IMRT plans. The maximal dose in the IMRT plan was ≤105% of the prescribed dose; the ipsilateral lung (V_20_, volume of lung receiving >20 Gy) was ≤25% and the heart was V_50_ ≤50% in patients with left breast cancer. The prescription doses for both plans are as follows: total dose 5000 cGy/25,200 cGy/times, five times per week, a total of 25 times.

### Statistical analysis

Dosimetric comparisons between the tumors and OARs were completed based on the following parameters from a dose-volume histogram (DVH): D_95_, maximal dose at 95%; D_max_, dose received by ≤1% volume of the PTV; D_min_, dose received by ≥99% volume of the PTV; and D_mean_ of the PTV, V_30_, V_20_, V_10_ and V_5_ of the lung (fraction of the lung volume receiving >30, 20, 10 or 5 Gy, respectively) and V_30_, V_40_ and V_50_ of the heart (fraction of the heart volume receiving >30, 40 or 50 Gy, respectively) in patients with left breast cancer. Statistical analyses were performed using SPSS, version 16.0 (SPSS. Inc., Chicago, IL, USA). Student’s paired, two-tailed t-tests were used to determine statistical significance. P≤0.05 was considered to indicate a statistically significant difference.

## Results

### Dosimetric comparison between the PTV in IMRT and CR

An adequate dose coverage of the mammary glands and lymph nodes in the IMRT and CR plans was achieved in the majority of patients. For example, 95% of the PTV of the mammary glands was delivered by ≥95.4% of the prescribed dose. Similarly, the CR PTV was 95% in the CR plan, as the partial PTV was located in a low-dose region of the tangential fields. Of note, the volume of the low-dose region in the breast PTV was observed to correlate with the breast tissue thickness at the medial beam edge of the tangential fields.

**Figure 1:**
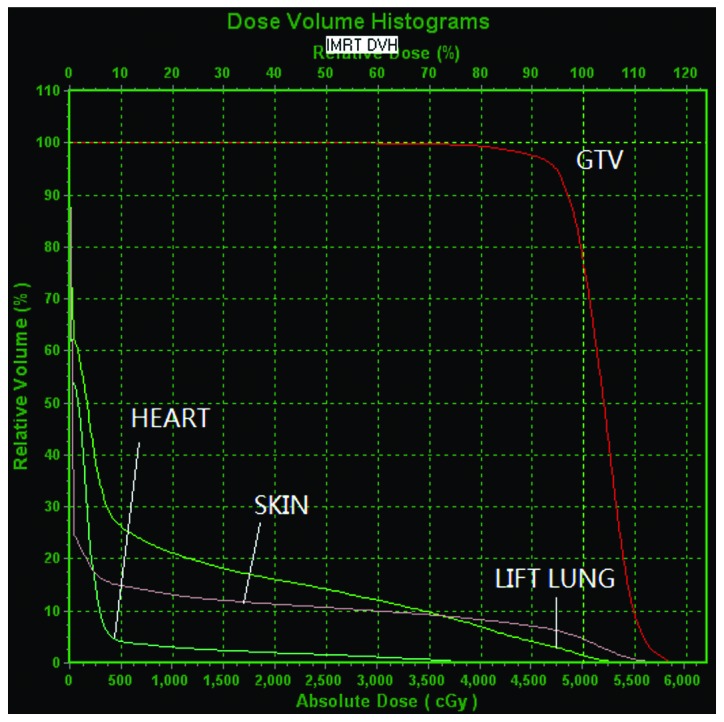
DVH of IMRT for a typical patient, showing results for the ipsilateral lung, heart and skin. DVH, dose-volume histogram; IMRT, intensity-modulated radiotherapy; GTV, gross tumor volume.

**Figure 2:**
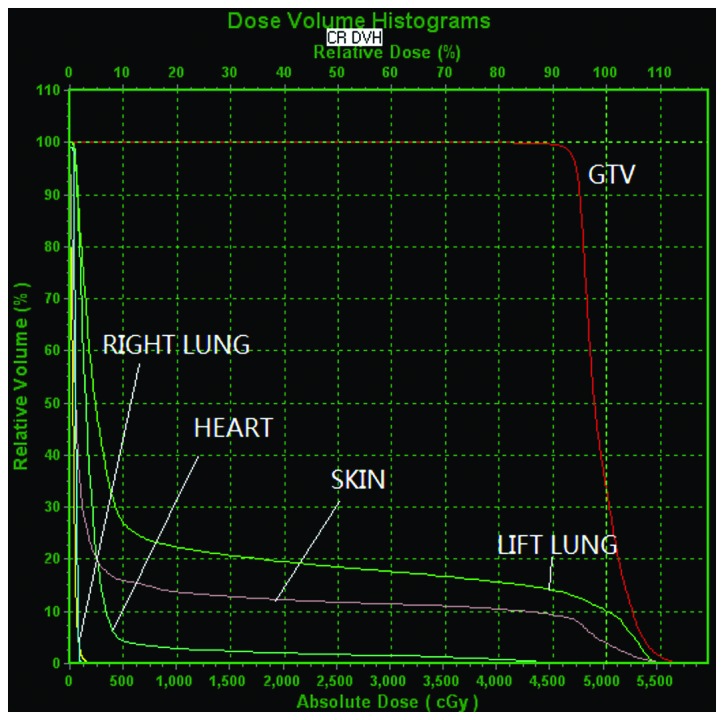
DVH of CR for a typical patient, showing results for the lungs, heart and skin. DVH, dose-volume histogram; CR, conventional radiotherapy; GTV, gross tumor volume.

**Figure 3:**
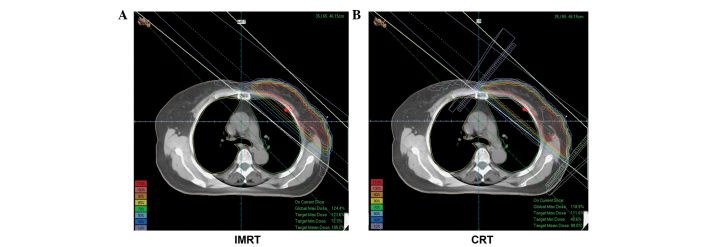
Isodose distributions from (A) intensity-modulated radiotherapy and (B) conventional radiotherapy for a typical patient.

DVH plots of the two modalities for the PTV of a typical patient are illustrated in Figs. 1 and 2. D_95_, D_max_, D_min_ and D_mean_ values are presented in Table I. No significant difference in D_mean_ was observed between the IMRT and CR (P=0.326). Furthermore, IMRT acquired a significantly lower D_max_ (P=0.015), but a higher D_min_ (P=0.031), as compared with the CR. Accordingly, the IMRT improved dosimetric homogeneity more efficiently, without dosimetric hot and cold spots (Fig. 3).

### Comparison between the dosimetric parameters of OARs

The dosimetric parameters of OARs, including V_5_, V_10_, V_20_ and V_30_ of the ipsilateral lung and V_30_, V_40_, V_50_ and D_mean_ of the heart are listed in Tables II and III, respectively. DVH plots for OARs of the two modalities are depicted in Figs 1. and 2. V_5_, V_10_, V_20_ and V_30_ of the ipsilateral lung were significantly reduced by 10.8, 8.4, 6.9 and 6.4%, (P=0.000, P=0.001, P=0.003 and P=0.002, respectively) in IMRT, as compared with the CR. Additionally, V_30_ of the heart was significantly reduced (P=0.046) and decreasing trends for V_40_ and V_50_ were observed in the IMRT. Collectively, the IMRT improved OAR protection by decreasing the irradiation dose and volume sparing the OARs, as compared with the CR.

## Discussion

Over the past decade, there has been a rapid rise in the application of advanced radiation delivery technologies for the curative management of numerous types of solid cancer. Clinical irradiation patterns have shifted from conventional 2D therapy to a more developed 3D therapy based on CT (4,19). Furthermore, 3D conformal radiation, including IMRT, exhibits accurate information with regard to the radiation dose to the affected breast, regional nodes and adjacent normal tissues. Therefore, this approach may reduce morbidity and improve long-term cosmesis, while maintaining local tumor control (20–22). In the three published randomized trials of IMRT in breast cancer (2,23,24), the focus was on early-stage breast cancer treatment and, thus, the radiation target volume was only the breast. In these trials, IMRT only improved the radiation dose homogeneity, which was due to the elimination of significant hotspots in the breast that presented in wedge-based 2D plans (23,24). Improved radiation dosimetry is associated with improvements in acute radiation reactions, including skin dermatitis and overall breast cosmesis (12,25,26). IMRT may be beneficial for the treatment of node-positive breast cancer, particularly when the internal mammary nodal regions require treatment (17,27,28). At the Department of Tumor Radiotherapy, The First Affiliated Hospital of Anhui Medical University, CR is the routine technique for patients receiving breast-conserving surgery. Larger irradiated volumes of the ipsilateral lung and heart have been identified in CR compared with IMRT, as well as larger fields of rectangular shape, formed by jaws in the X and Y direction. However, intensity gradients in CR are only generated in a single direction (?).

The advantages of IMRT have been discussed in several studies; however, the results remain contradictory (17,19). Dogan *et al* (28) demonstrated the benefits of the IMRT plan as compared with a standard treatment plan using optimized beam weights and wedges. However, the treatment plans were not compared with the rectangular tangential fields commonly used in clinical practice. Additionally, it has been demonstrated that standard dose optimization, also known as two-step IMRT (21,27,29), does not generate the optimum intensity distributions, due to degradation during the segmentation process. Therefore, the clinical application of IMRT for breast cancer remains unknown.

In the current study, although no significant difference in D_mean_ was identified between IMRT and CR (P=0.326), IMRT acquired a significantly lower D_max_ (P=0.015) and a higher D_min_ (P=0.031), which is consistent with previous reports (6,28,30,31). This suggested that IMRT improves dosimetric homogeneity and uniformity without dosimetric hot and cold spots. Furthermore, compared with CR, IMRT decreased the OAR volumes receiving high doses and increased the volumes receiving low doses. Additionally, CR increased the volumes exposed to the ipsilateral lung and heart compared with the IMRT. Accordingly, in order to obtain clinically accepted plans, the IMRT plan requires clinical planners with advanced treatment skills to achieve different combinations of wedge angles, collimator angles and beam weights.

IMRT has been used to avoid late toxicity, including pneumonitis, lung fibrosis and coronary heart disease (15). However, the probability for radiation-induced secondary malignancies may increase when larger volumes of normal tissue are exposed to lower doses. In the outcomes presented in this study, IMRT yielded a smaller proportion of irradiated volume in high-dose areas when compared with CR, but a larger proportion in low-dose areas. This was likely to be due to increased leakage from more segments in IMRT. To date, only skin reactions from reverse IMRT have been reported (26).

With regard to set-up uncertainty during the treatment process, the boundary of the irradiation fields may be expanded far enough to ensure the PTV is completely included when using CR and IMRT techniques. However, set-up accuracy in IMRT must be the focus, which may be improved through breath gating and imaging guidance techniques. Notably, the lymph nodes were not included in the treatment volume in this study. In addition, with the limited sample size, clinically meaningful improvements with the use of IMRT require further, large randomized trials.

In conclusion, CR has exhibited satisfactory results in breast cancer patients treated with breast-conserving surgery in our department at The First Affiliated Hospital of Anhui Medical University. However, the present dosimetric analyses demonstrated that IMRT provides improved uniformity and coverage of target volumes and an associated reduction of dose delivery to critical organs when compared with CR. In addition, IMRT decreased the OAR volumes receiving higher doses and increased the volumes receiving lower doses. Clinical trials and long-term follow-up may be required to evaluate the clinical significance of the dosimetric characteristics associated with IMRT.

## Figures and Tables

**Table I:** Comparison between the D_95_, D_min_, D_max_ and D_mean_ of the PTV in IMRT and CR.

Variables	D_95_	D_min_, cGy	D_max_, cGy	D_mean_, cGy
CR	4518.3±60.4	3807.9±243.6	5832.2±61.4	5086.9±49.0
IMRT	4541.4±35.4	3868.4±248.3	5795.0±54.5	5075.8±47.3
P-value	0.009	0.031	0.016	0.326

D_95_, maximal dose at 95%; D_min_, minimum dose; D_max_, maximum dose; D_mean_, mean dose; PTV, planning tumor volume; IMRT, intensity-modulated radiotherapy; CR, conventional radiotherapy.

**Table II:** Comparison between the dosimetric parameters of the ipsilateral lung.

	Ipsilateral lung			
	
Variables	V_5_, %	V_10_, %	V_20_, %	V_30_, %
CR	38.3±0.8	31.8±0.8	27.7±0.9	24.9±1.0
IMRT	27.5±1.7	23.4±2.0	20.8±2.0	18.5±2.0
P-value	0.000	0.001	0.003	0.002

CR, conventional radiotherapy; IMRT, intensity-modulated radiotherapy. V_n_ the volume of the lung when the patient received a dose of nGy of radiation.

**Table III:** Comparison between the dosimetric parameters of the heart in patients with left breast cancer.

	Heart			
	
Variables	V_30_, %	V_40_, %	V_50_, %	D_mean_, cGy
CR	10.06±1.7	4.13±1.0	1.3±0.5	598.4±118.2
IMRT	5.3±1.4	1.9±0.5	0.0±0.0	348.3±91.6
P-value	0.046	0.095	0.076	0.004

D_mean_, mean dose; CR, conventional radiotherapy; IMRT, intensity-modulated radiotherapy. V_n_ the volume of the lung when the patient received a dose of nGy of radiation.
